# Editorial: *Aegilops*: Promising Genesources to Improve Agronomical and Quality Traits of Wheat

**DOI:** 10.3389/fpls.2020.01060

**Published:** 2020-07-14

**Authors:** Marianna Rakszegi, István Molnár, Éva Darkó, Vijay K. Tiwari, Peter Shewry

**Affiliations:** ^1^Agricultural Institute, Centre for Agricultural Research, Martonvásár, Hungary; ^2^Plant Science and Ladscape Architecture, University of Maryland, Washington, MD, United States; ^3^Department of Plant Science, Rothamsted Research, Harpenden, United Kingdom

**Keywords:** *Aegilops* sp., stress tolerance, quality traits, genome analysis, alien introgression

## Introduction of Aegilops

*Aegilops* species have contributed significantly to wheat improvement despite the challenges in exploiting wild species, such as crossability and incompatibility (Börner et al., 2015; Fedak, 2015). They have been used in particular as sources of genes conferring resistance to biotic stresses, but also for more complex traits such as abiotic stress and yield.

The genus *Aegilops* consists of 22 species with the C, D, M, N, S, T and U genomes, which have high allelic diversity relative to wheat. *Aegilops tauschii*, the D-genome donor of bread wheat, has been most widely used for wheat breeding, followed by *A. speltoides* and *A. ventricosa*. However, because most *Aegilops* species are in the secondary and tertiary gene pools of wheat they are difficult to utilize due to recombination barriers and useful variation from these species is only available in the form of translocation/introgression lines.

## Identification of Diversity In Traits For Wheat Improvement

As sources of tolerance to **biotic stresses**, 20% of the total number (over 75) resistance gene loci identified in cereals are present in *Aegilop*s species (Ponce-Molina et al., 2018). These include two thirds of the 54 loci for resistance to powdery mildew (Tang et al., 2018), and the 12 resistance loci for Cereal Cyst Nematodes (Ali et al., 2019). In the present topic, the addition of *A.markgrafii* chromosomes to wheat increased the resistance to 19 of 20 powdery mildew isolates in addition line AV(E) (Niu et al.).

New stem rust resistance genes have also been identified in *Aegilops*, such as *Sr46*, *Sr47*, *Sr51* and *Sr53* (in *A. tauschii*, *A. triuncialis*, *A. searsii* and *A. geniculata respectively;* Liu et al., 2011a; Liu et al., 2011b; Klindworth et al., 2012; Yu et al., 2015) and three additional genes in *A. tauschii* (Rouse et al., 2011), three genes in *Ae. sharonensis* (Singh et al., 2015; Yu et al., 2017) and one gene in *A. umbellulata* (Edae et al., 2016). In addition, it has been reported that 81% of accessions of *A. longissima*, 94% of *A. neglecta* and 88% of *A. cylindrica* (DDCC) and *A. peregrina* (SSUU) were resistant to the Ug99 race group of the stem rust pathogen (*Puccinia graminis* f. sp. tritici) (Huang et al., 2018; Olivera et al., 2018) (Kishii). In this topic Niu et al. reported that wheat/*A.markgrafii* addition lines AII(C) and AIII(D) were resistant to Ug99. Furthermore, *A. biuncialis, A. caudata, A. comosa, A. cylindrica, A. geniculata, A. neglecta, A. peregrina, A. triuncialis, and A. umbellulata* were evaluated for resistance to three highly virulent races (TTKSK, TRTTF and TTTTF) of *P. graminis* f. sp. tritici with 60–70% exhibiting low infection types. Association analyses showed that for a given species, the resistance genes are effective against multiple races (Olivera et al., 2018).

Brisco et al. (2017) identified several *A. tauschii* accessions showing resistance to Fusarium Head Blight and studies reported in this topic (Szabo-Hever et al.) have shown that *A. tauschii* accessions decreased disease severities by 18.3%, suggesting that either the D genome or the increased ploidy level could contribute to resistance in synthetic hexaploid lines.

*Aegilops* species are also a resource for novel genes and alleles providing tolerance to **abiotic stresses**. In this topic Suneja et al. provide a good example of the identification of several *A. tauschii* accessions as potential donors of adaptive plasticity to stress.

*Aegilops* species also serve as a resource for introducing useful genetic variation in **grain processing and nutritional quality** in wheat (*Triticum aestivum*). Seed storage proteins are the major determinants of end product quality and mainly consist of glutenins and gliadins. A large number of allelic forms of these proteins have been identified in *Aegilops* species and in some *Aegilops* species such as *A. searsii, A. geniculata* and *A. longissima* this variation have been linked with improved breadmaking quality. *Aegilops* species has also been explored for diversity in the grain texture-related proteins, called puroindolins (Pins) and grain softness proteins (GSP). In particular, studies carried out in a number of countries have identified almost 100 alleles of *Pin a, Pin b* and *GSP* across 200 lines/accessions. This allelic variation could be utilized in breeding programs to extend the textural characteristics of wheat (Kumar et al.).

*Aegilops* has attracted further attention in relation to increasing the grain mineral content of wheat. In particular, to produce biofortified wheat with higher the contents of iron and zinc in order to alleviate deficiencies in these minerals which currently affect more than 2 billion people worldwide (Cakmak, 2017; Black et al., 2013; Velu et al., 2018b). Some *Aegilops* species have been reported to contain three to four-fold higher concentrations of Zn and Fe grain content than wheat, including *A. longissima* (Sl), *A. kotschyi* (US), *A. peregrina* (US), *A. cylindrica* (CD), *A. ventricosa* (DN) and *A. geniculata* (UM) (Rawat et al., 2009). Amphiploid lines of durum wheat with *A. longissima*, partial amphiploids of bread wheat with *Ae. kotschyi* and addition/substitution lines of bread wheat with *A. kotschyi* also showed two to three times higher concentrations of Zn and Fe in grain than the wheat checks (Tiwari et al., 2008; Tiwari et al., 2010; Rawat et al., 2011), indicating that they are promising resources to improve wheat composition. Velu et al. developed translocation lines with rye and different *Aegilops* species in a wheat genetic background to increase the Zn content. Although the potential health benefits of *Aegilops* species by increased minerals in wheat have not yet been realized, they should have an impact in the future (Kishii).

## Establishment and Exploitation of Genomic Resources In *Aegilops* spp.

A **high-throughput genotyping platform** has been specifically designed for screening species related to wheat and used to screen multiple accessions representing all species in the genus *Aegilops*. This application was useful for identifying diversity and determining the relationships within and between *Aegilops* species (Przewieslik-Allen et al.). Genome adaptability to environmental changes, especially to rapid climatic fluctuations, underlies the survival and **evolution of species**. In wild species, genetic and epigenetic changes are accompanied by significant alterations in the complex nuclear repetitive DNA fraction. Perpetual intra-organismal reshuffling of repetitive DNA mirrors the structural plasticity of the *A. speltoides* genome, which is related to genetic diversity through the distribution of the species in contrasting ecogeographical environments (Pollak et al.). Ruban and Badaeva proposed a model for the **evolution of the S-genome** of *A. speltoides*. The genomes of allopolyploid wheats have evolved by different species-specific chromosome translocations, sequence amplification, and elimination and re-patterning of repetitive DNA sequences. These events occurred independently in different wheat species and in *A. speltoides*. The 5S rDNA locus of chromosome 1S was probably lost in ancient *A. speltoides* prior to formation of cultivated *Triticum timopheevi* (AAGG genomes), but after the emergence of ancient emmer (AABB genomes). rDNA profiling and distribution was used to divide diploid *Aegilops* species into two groups corresponding to the Emarginata and Truncata sub-sections. It was found that the evolution of Emarginata species was associated with an increase of C-banding and heterochromatin, amplification of Spelt-52, re-pattering of the pAesp_SAT86, and a gradual decrease in the amount of the D-genome-specific repeats pAs1, pTa-535, and pTa-s53.

*A. tauschii* (2n = 2x = 14, genome DD), also known as Tausch’s goatgrass, is the D genome donor of hexaploid bread wheat (*T. aestivum*, 2n = 2x = 42, AABBDD genome). It is a rich source for tolerance to biotic and abiotic stresses. A **TILLING** (Targeting Induced Local Lesions In Genomes) population of *A. tauschii* (TILL-D) was developed using ethyl methanesulphonate (EMS) as a mutagen which, together with the newly published *A. tauschii* reference genome sequence, will facilitate the discovery and validation of genes for agronomically important traits and their transfer into bread wheat (Rawat et al.). Population structure analysis based on high quality SNPs confirmed the differentiation of *A. tauschii* into two lineages (L1 and L2). A **MiniCore collection** consisting of 29 L1 and 11 L2 accessions was identified based on genotypic, phenotypic and geographical data. This captures 84% of the total allelic diversity in the whole collection, showing that it is possible to reduce the number of accessions which need to be screened by 90% (Singh et al.). A **genome wide association study (GWAS)** of the grain Fe, Zn, Cu and Mn contents also indicated that *A. tauschii* lineage 2 had higher Fe and Cu concentration than lineage 1 (Arora et al.). The associations were related to genes encoding transcription factor regulators, mineral transporters and phytosiderophore synthesis.

The **stability of translocation or alien introgression** lines is always of concern. King et al. developed homozygous wheat/*A. muticum* dihaploid introgression lines and characterized their stability using genomic *in situ* hybridization and SNP analysis (King et al.). Zhang et al. studied the **efficiency of transferring** *A. tauschii* segments to wheat using a synthetic octaploid (AABBDDDD, 2n = 8x = 56) and used bridge crosses to mapped QTL for agronomically important traits.

Wheat/*A. markgrafii* **disomic addition lines** carrying the chromosomes B, C, D, E, F and G, respectively, were screened with SSR markers showing that they corresponded to wheat homoeologous groups 2, 5, 6, 7, 3, and 4, respectively. **Useful markers** were also identified for chromosome engineering of wheat (Niu et al.).

The papers brought together in this topic therefore illustrate the range of current research on the charcterisation of *Aegilops* species and identification of important traits for exploitation in wheat improvement.

## Author Contributions

MR, PS, VT, ÉD, and IM wrote this manuscript.

## Funding

The topic is based upon the workplan of the COST Action 18101 SOURDOMICS—Sourdough biotechnology network towards novel, healthier and sustainable food and bioprocesses, where the members of the working group 1 were supported by COST (European Cooperation in Science and Technology). COST is a funding agency for research and innovation networks.

## Conflict of Interest

The authors declare that the research was conducted in the absence of any commercial or financial relationships that could be construed as a potential conflict of interest.
